# The Future of the Pharmacist

**Published:** 1911-12

**Authors:** 


### BUFFALO MEDICAL JOURNAL

**A Monthly Review of Medicine and Surgery**

*EDITOR*

A. L. BENEDICT.

All communications, whether of a literary or business nature, books for reviewand exchanges, should be addressed to the editor, 354 FRANKLIN STREET, BUFFALO, N. Y. Please make personal or telephonic calls before 1 p. m

THE BUFFALO MEDICAL JOURNAL will publish regularly, full transactions of any professional organization in western New York at cost. Personal notices, brief reports of society proceedings, and the like, are always welcome and will be published without charge.

Subscriptions may commence at any time. $2.00 per year in advance,

VOL. LXVII. DECEMBER, 1911. No. 5

*Will your biography be written in verbs or adjectives?*

### The Future of the Pharmacist

HE first issue of *Successful Medicine,* a bright, interesting journal devoted to the business side of medicine, and edited by H. R. Harrower of Chicago, contains an article the gist of which is that, "It pays to dispense." We do not need to recapitulate the arguments on this matter. In a narrow sense and under certain local conditions, it does pay to dispense, just as it pays to prescribe over the counter. In the broad sense of the ultimate welfare of all parties concerned, it does not pay for any man to do that for which he is not specially trained and to take some other man's business from him.

With the present overcrowded conditions of both the medical and the pharmaceutic profession, and the growing scarcity of money in proportion to its purchasing power, it is inevitable that the keen competition for a living should result in violations of ethical principles, among the members of each profession and that the effects should be felt within and across professional lines. But we must maintain our ideals and make the economic conditions fit the ideals instead of lowering our ideals to fit an economic disturbance of supply and demand which can be corrected. This is especially true because, however much the individual may gain in a low financial sense from disregarding principles of ethics, no lowering of standards will produce any genuine improvement.

There are certain details, however, in which common sense —which is always good ethics—requires a readjustment of standards. First of all, we must face the fact that the modern materia medica contains a great many valuable remedies and a great many elegant and convenient preparations of old drugs, which can not be manufactured in a retail drug store. The pharmacist must, therefore, act as the retailer of package goods. In the country and, to some extent in the city, economy, convenience and promptness of supply, compel the physician to dispense to a greater or less degree.

Secondly, the time-honored liquid mixture of principal drug, corrigent, adjuvant, and vehicles, has very largely given place to condensed medication in which tastelessness, directly or indirectly secured, has proved superior to older nations of palatability, and in which one drug is given at a time, for its own effect, in a dose that may be regulated from time to time without regard to that of adjuvants.

Thirdly, even within the memory of physicians still in active practice, drugs have gradually given place to mechanic, electric and other imponderable therapeutic agents and such drugs as are used, are being applied locally by the physician himself.

We do not mean to imply a disbelief in drugs. Many diseases are distinctly perversions of bodily chemistry; obviously they demand chemic correction. Moreover, while the ultimate explanation is not known, the fact remains that many drugs produce mechanic and chemic effects on various vital organs. We are inclined to believe that, while many drugs have been discarded and while we have learned that the use of others has been based on misconceptions or may properly yield to other measures, at no time have drugs, properly prepared and used with discrimination, produced such satisfactory results as at present.

But, the fact must be faced squarely that the old use of complicated prescriptions has been greatly curtailed and that it must continue to decline. There are two ways of solving the problem for the druggist. The easiest way is for him to relegate his profession to still greater obscurity, pushing his prescription counter farther and farther to the rear, and making it smaller and smaller, giving greater and greater attention to the business of soda water, cigars, magazines, postal, express, gas, laundry and other agencies, stationery and toilet articles and the like. Some druggists have already solved the problem so satisfactorily in this way that they say openly that they do not care an obstruction to a stream of water-n, whether they get prescriptions or not. This is an undignified solution but a few weeks of Euroean travel rather tend to convert one to the view that the type of American drug store, with its many conveniences, is something to be retained, even with its quack medicines. The latter, indeed, have a conservative value in acting as fool killers and in reducing certain patients to the point at which they are glad to accept skilled medical attendance.

The second way of solving the problem is for the druggist to study critically the advance of medical science and art and to meet the new demands made upon him or, rather, which may be made upon him if he will prepare for them. For example, he might secure quite a little business merely by keeping track of new remedies, inquiring as to the liklihood of their use in his own community, putting them in stock or, at least arranging for their delivery more promptly than the physician can secure them on direct order. Co-operation would help in this regard.

Another development of pharmaceutic demand is well illustrated by salvarsan. This is the year 1911. More is required of almost every profession and trade in this year than in 1811, vastly more than in 1711. It ought to be possible to send a prescription for salvarsan, ready for use, to any drug store, in 1911, with the same confidence of its prompt and proper preparation, as for laudanum in 1811. Einhorn's heads and packets for intestinal tests belong in the same category. Salol and keratin coated pills are another example. Suppositories and bougies of gelatin, cocoa butter, etc., are another. The fact that expensive and troublesome apparatus for preparing salvarsan are advertised in medical journals to physicans shows that the pharmacist is blinding his eyes to a proper and ultimately profitable extension of his scope.

The words pharmacist and chemist used to be almost synonymous. Can the physician telephone to his druggist for a decinormal solution of sodium hydrate, or a 1 per cent. solution of a reagent or drug for local use, and be sure of accuracy? Some time ago, we wrote a prescription for a certain extract, in definite amount. We weighed it in its container, soaked it in alcohol, dried and weighed the box and label, subtracted, and found an error of 25 per cent.

Has the druggist even weights and scales and graduates of sufficient accuracy for even approximate clinical determinations? Can he analyze urine in a suspected case for arsenic or morphine —fairly simple chemic examinations? Can he prepare extemporaneously, chromic catgut, aseptic dressings, etc?

We are writing in no fault-finding mood. We merely wish to impress on the pharmaceutic profession that there is a vast field of usefulness and profit requiring a development of special skill, along logical lines and into which it is practically forcing the medical profession to enter. And, meantime, the former profession is complaining of the non-support of the latter and trying to entice him back by a display of elixirs and 4-ounce mixtures which are almost out of date and which any one with a fairly accurate balance and a 25 cent graduate can compound.

